# Visual Function in Athletes from Different Team Sports and Non-Athlete Controls

**DOI:** 10.3390/life15101619

**Published:** 2025-10-16

**Authors:** Henrique Nascimento, Ana Roque, Clara Martinez-Perez

**Affiliations:** 1 Instituto Superior de Educação e Ciências de Lisboa (ISEC Lisboa), Alameda das Linhas de Torres, 179, 1750-142 Lisboa, Portugal; henrique.nascimento@iseclisboa.pt (H.N.); ana.roque@iseclisboa.pt (A.R.); 2 Applied Physics Department (Optometry Area), Facultade de Óptica e Optometría, Universidade de Santiago de Compostela, 15705 Santiago de Compostela, Spain

**Keywords:** visual acuity, team sports, peripheral vision, basketball, roller derby

## Abstract

Visual skills are increasingly recognized as critical to athletic performance, yet it remains unclear whether participation in specific team sports is associated with enhanced visual function. This cross-sectional study compared visual acuity, peripheral vision, stereoacuity, ocular alignment, and refractive error among 52 participants aged 15–56 years: basketball (n = 10), futsal (n = 9), hockey (n = 12), roller derby (n = 9), and non-athlete controls (n = 12). Standardized assessments included best-corrected visual acuity (logMAR), Hirschberg shift, peripheral perception using a tachistoscope, stereoacuity with the Randot^®^ test, and non-cycloplegic autorefraction. Group comparisons were conducted using ANOVA, post hoc analyses, and regression models adjusted for age. Significant differences were observed only for visual acuity (F(4, 47) = 4.46, *p* = 0.003, η^2^ = 0.275): non-athlete controls (0.00 ± 0.08 logMAR) and basketball players (0.02 ± 0.05) showed the best performance, while roller derby athletes demonstrated the poorest (0.16 ± 0.12). No significant group differences were found for peripheral vision, stereoacuity, Hirschberg deviation, or refractive error, and the poorer acuity in roller derby remained after adjustment for age. These findings suggest that participation in team sports does not universally confer superior visual function and that static clinical measures may overlook the dynamic visual–motor strategies that underlie athletic performance.

## 1. Introduction

Sport vision (SV) is a multidisciplinary specialty that has gained significant attention over the past two decades, recognizing that visual skills are essential to athletic performance beyond traditional measures of strength, speed, and endurance [1]. Numerous studies have shown that athletes with superior visual processing and reaction speed can anticipate play and make faster decisions, often distinguishing elite performers from their peers [2,3]. This has led to the integration of vision assessment and training in athlete development programs, from early eye examinations to the routine inclusion of visual tests in Olympic competitions [3,4].

SV is not limited to visual acuity or clarity of sight; it also includes the efficiency with which the brain processes visual information, as well as the coordination of sensory and motor systems [5]. The European Academy of Sport Vision (EASV) promotes a multidisciplinary approach to maximizing visual system function, tailored to the demands of specific sports [3]. Core visual skills relevant in sports include static and dynamic acuity, peripheral vision, spatial focus, depth perception (stereopsis), and eye–limb coordination [3,6]. Among these, stereopsis is particularly important for three-dimensional spatial analysis and interaction with dynamic environments [6].

The interaction between visual and locomotor systems is also crucial, as vision guides and adapts movement strategies in response to environmental complexity [7,8]. For instance, research shows that reduced stereopsis, whether due to occlusion, monocular blur, or amblyopia, impairs hand–eye coordination and alters movement patterns, highlighting the essential role of binocular and peripheral vision [9,10,11].

Visual demands vary greatly across sports and roles. For example, football officials make over 130 decisions per match, often under time pressure and in variable environmental conditions [12]. Elite referees and athletes have demonstrated superior skills such as dynamic visual acuity, peripheral awareness, saccadic eye movements, and recognition speed compared to less experienced individuals [13,14]. In addition to the “hardware” of vision (acuity, basic abilities), expert performers excel in the “software” of visual strategies, such as anticipation, scanning efficiency, and decision-making [15,16]. Peripheral vision is a key attribute in both everyday life and sports, enabling athletes to monitor the environment, opponents, and teammates outside the direct line of sight [17]. Research and clinical cases have highlighted how peripheral input is vital for scene understanding, spatial awareness, and effective responses in complex environments [18].

Despite the growing body of evidence, most studies focus on single sports or elite performers, with few directly comparing visual skills across different sporting contexts or including non-athlete controls. Furthermore, confounding factors such as age or refractive status are often underexplored, and it is not known whether participation in specific sports always leads to superior or merely distinct visual abilities [1,19]. There remains a clear need for comparative research to systematically evaluate the visual profiles of athletes from different sports, using well-defined controls and accounting for key demographic variables.

In the present study, the term “athlete” refers to individuals actively engaged in structured, competitive training and participation for at least one year in their respective sport. This inclusive definition was chosen to represent amateur and semi-competitive players, reflecting the diversity commonly encountered in applied sports vision contexts. Previous studies have adopted similar criteria when assessing visual function among non-elite athletes [1,19]. The sports selected—basketball, futsal, hockey, and roller derby—were chosen because they represent visually demanding, fast-paced team disciplines requiring constant visual monitoring of the environment, rapid decision-making, and peripheral awareness, while differing in tempo, spatial constraints, and contact intensity.

The objective of the present study is to systematically assess visual function (including visual acuity, peripheral vision, stereoacuity, and refractive status) in athletes from basketball, futsal, hockey, roller derby, and a non-athlete control group. By directly comparing these groups and controlling for age, this research aims to clarify whether participation in specific sports is associated with distinct or enhanced visual profiles—or if, in some cases, athletes may not differ from or may even underperform compared to non-athletes. These findings may guide future screening and training strategies in sport vision.

## 2. Materials and Methods

### 2.1. Study Design and Participants

This cross-sectional, observational study aimed to comprehensively evaluate visual function across different athlete groups and non-athlete controls. Data collection was conducted at the Sports Vision High-Performance Laboratory of Instituto Superior de Educação e Ciências (ISEC Lisboa, Lisbon, Portugal) between April and June 2024. Athlete participants were recruited through direct contact with local sports clubs and university teams, while control participants were recruited via institutional announcements and voluntary response to an open call distributed at ISEC Lisboa.

A total of 52 participants were included: basketball (n = 10), futsal (n = 9), hockey (n = 12), roller derby (n = 9), and non-athlete controls (n = 12). No formal a priori sample size calculation was performed; the sample size was determined by feasibility and athlete availability across the included sports. To aid interpretation, effect sizes and 95% confidence intervals were reported.

Eligibility criteria for athletes included (1) being an active participant in competitive training and competition for at least one year in the specified sport (at amateur or semi-professional level), ensuring regular engagement in structured practice and competition; (2) age between 15 and 56 years to include physically active individuals while minimizing the influence of age-related ocular changes; and (3) no known neurological or systemic disease affecting vision. Control participants were required to have no regular participation in organized sports or competitive training within the last 12 months and were also asked about informal or non-organized physical activity (e.g., jogging, cycling, or gym exercise); individuals engaging in such activities more than three times per week were excluded to maintain a non-athlete control group. Exclusion criteria for all groups were (1) history of ocular trauma or surgery, (2) use of medication affecting visual or cognitive performance, or (3) uncorrectable visual impairment (best-corrected VA worse than 0.2 logMAR in either eye).

The study followed the ethical standards of the Declaration of Helsinki and was approved by the Ethics Committee of the Higher Institute of Education and Sciences of Lisbon (ISEC Lisbon) on 11 March 2024 (approval ID: CE/2024/03/11). All participants provided written informed consent. All data were anonymized and stored securely. The research adhered strictly to the ethical principles outlined in the Declaration of Helsinki

### 2.2. Visual Function Assessment

All visual function assessments were performed in person by a licensed eye care professional (optometrist) with expertise in sports vision, using standardized and calibrated equipment in a quiet, well-lit environment at the Sports Vision High-Performance Laboratory of ISEC Lisboa. A preliminary ocular health screening, including anterior and posterior segment evaluation with direct ophthalmoscopy, was conducted to exclude any participant with ocular pathology or visual impairment (best-corrected VA worse than 0.2 logMAR). The assessment protocol was identical for all participants to ensure methodological consistency. Visual acuity was measured monocularly and binocularly using a standardized logMAR chart (ETDRS or equivalent) positioned at a distance of four meters under photopic lighting conditions. Participants who regularly used optical correction were assessed with their usual prescription. For analysis, the binocular logMAR value with the best correction was recorded to allow reliable comparisons between groups. Ocular alignment was assessed using the Hirschberg corneal light reflex test and recorded using photorefractometry, which quantifies displacement in degrees (°), providing an objective estimate of latent or manifest strabismus or phoria. Peripheral perception was assessed using a digital tachistoscope. During this test, participants were instructed to fixate centrally while visual stimuli were presented simultaneously in each of the four visual quadrants. The number of stimuli detected in each presentation was documented, and these values were calculated as the percentage of correct responses across all presented objects to obtain a composite peripheral vision score for each participant. Although this method does not correspond to static automated perimetry, it provides a functional measure of peripheral awareness under dynamic conditions, offering an ecologically valid assessment relevant to sports performance. Visual stereoacuity was determined using the Randot^®^ stereo test (Stereo Optical Co., Inc., Chicago, IL, USA), administered at a distance of 40 cm under standard lighting. The smallest binocular disparity correctly identified by the participant was recorded in seconds of arc, reflecting their ability to perceive depth. Refractive error was measured with non-cycloplegic autorefraction using a Zeiss Profiler Plus autorefractometer, and the spherical equivalent was calculated for each eye by adding the sphere value and half the cylinder value, with the final value representing the average of both eyes. All assessments were completed in a single session lasting approximately 30 to 40 min per participant. To minimize potential confounding factors, all individuals were instructed to abstain from caffeine and strenuous physical activity for at least two hours prior to testing. Athletes were specifically scheduled for assessment on non-competition days and after a minimum of twelve hours of rest following their last training session or competitive event.

### 2.3. Statistical Analysis

All statistical analyses were performed using R (version 4.4.2). Descriptive statistics (means, standard deviations, medians, and 95% confidence intervals) were calculated by group. Normality was assessed with the Shapiro–Wilk test and homogeneity of variances with Levene’s test. Group comparisons were conducted using one-way ANOVA with Tukey’s HSD post hoc test when assumptions were met; otherwise, the Kruskal–Wallis and Dunn’s tests (with Benjamini–Hochberg correction) were applied. Differences between athletes and controls were examined using *t*-tests or Wilcoxon rank-sum tests as appropriate. Correlations were analyzed using Pearson’s or Spearman’s coefficients. Multiple linear regression was used to evaluate the independent effects of sport group and age on visual acuity. Effect sizes (partial eta squared and Cohen’s d) were reported. Statistical significance was set at *p* < 0.05.

## 3. Results

**Figure 1:**
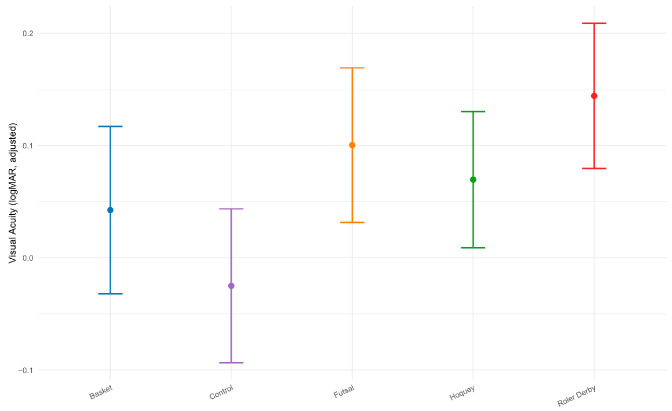
Estimated marginal means of visual acuity by sport.

The final sample comprised 52 participants aged 15 to 56 years (mean ± SD: 28.6 ± 8.4). Descriptive statistics and overall group comparisons (ANOVA *p*-values) for all visual variables are shown in Table 1. For VA, the ANOVA indicated a significant effect of sport type, F(4, 47) = 4.46, *p* = 0.003, with a large effect size (η^2^ = 0.275). On average, roller derby athletes showed the lowest VA scores (0.16 ± 0.12; note that lower logMAR values indicate better VA), while control participants (0.00 ± 0.08) and basketball players (0.02 ± 0.05) showed the highest. Post hoc comparisons using Tukey’s HSD confirmed that VA in roller derby athletes was significantly poorer than in both controls (*p* = 0.0036) and basketball players (*p* = 0.0152). Estimated marginal means adjusted for age are displayed in Figure 1.

Age also showed a significant difference across groups, F(4, 47) = 10.64, *p* < 0.0001, with the largest effect size observed among all variables (η^2^ = 0.475). Control participants were the oldest group (35.64 ± 10.88), while basketball (20 ± 3.97) and futsal players (24.89 ± 4.40) were the youngest. No statistically significant group differences were observed for Hirschberg deviation, peripheral vision, stereoacuity, or refractive error (all *p* > 0.05). Post hoc Tukey’s tests for Hirschberg deviation, peripheral vision, stereoacuity, and refractive error revealed no significant pairwise differences between sport groups (all adjusted *p* > 0.05). Only visual acuity (AV_logMAR) showed significant differences, specifically between roller derby and both control (*p* = 0.004) and basketball (*p* = 0.015) groups.

### Athlete vs. Non-Athlete Comparison

To explore potential differences between athletes and non-athletes, participants were regrouped into two categories: competitive athletes (basketball, futsal, hockey, and roller derby) and controls. Independent samples *t*-tests revealed that athletes exhibited significantly worse visual acuity compared to non-athletes (t(22.17) = 2.87, *p* = 0.009). However, this difference was primarily driven by the roller derby group, which showed the poorest VA among all participants, whereas other athlete groups performed comparably to controls.

No significant differences were found between athletes and non-athletes in Hirschberg deviation, peripheral vision, stereoacuity, or refractive error (all *p* > 0.1). However, a significant difference in age was observed, with athletes being younger than controls (t(12.01) = –2.61, *p* = 0.023). This raised the possibility that age could confound the observed differences in visual acuity.

To address this concern, a multiple linear regression model was constructed with logMAR as the dependent variable and both sport group and age as predictors. The model was statistically significant (F(5, 46) = 4.02, *p* = 0.004), with an adjusted R^2^ of 0.23, indicating that approximately 23% of the variance in visual acuity was explained by the predictors. Within the model, age was not a significant predictor (*p* = 0.175), whereas roller derby emerged as a marginally significant positive predictor of logMAR (β = 0.102, *p* = 0.064), suggesting that athletes in this group had poorer VA. A follow-up model including an interaction term between sport and age showed no significant interaction effect (*p* = 0.090), indicating that the relationship between sport and VA was consistent across age.

These findings suggest that sport-related differences in visual acuity are not attributable to age alone. Estimated marginal means adjusted for age confirmed that roller derby athletes consistently demonstrated poorer visual acuity than all other groups. Pairwise comparisons showed that, even after controlling for age, significant differences remained between roller derby and both control (*p* = 0.0036) and basketball (*p* = 0.0152) groups.

Effect size analyses further supported these results. Cohen’s d values indicated large differences in VA between several key comparisons: control vs. roller derby (d = 1.70), control vs. futsal (d = 1.26), and basketball vs. roller derby (d = 1.02).

No significant correlations were found between age and visual acuity across the entire sample (r = 0.19, *p* = 0.17), nor between refractive error and peripheral vision (Spearman’s ρ = –0.05, *p* = 0.75). Within individual sport groups, no consistent associations emerged between age and any visual variable.

Assumptions of homogeneity of variances were met for all variables (Levene’s *p* > 0.59). The normality assumption for residuals was marginally violated for visual acuity (Shapiro–Wilk *p* = 0.0001); therefore, non-parametric Kruskal–Wallis and Dunn’s post hoc tests were also conducted, confirming the pattern of significant differences observed in the ANOVA.

## 4. Discussion

This cross-sectional comparative study systematically examined visual function profiles in athletes from multiple team sports (basketball, futsal, hockey, and roller derby) relative to non-athlete controls, focusing on visual acuity, peripheral vision, stereoacuity, ocular alignment, and refractive error. While it is widely assumed that athletic participation universally enhances visual performance, our findings challenge this notion: only visual acuity (VA) showed significant group differences (see Table 1 and Figure 1), and unexpectedly, non-athlete controls and basketball players demonstrated the best acuity, whereas roller derby athletes exhibited significantly poorer VA, even after controlling for age. No differences emerged across groups for peripheral vision, stereoacuity, Hirschberg deviation, or refractive error (Table 1). These results both echo and diverge from recent findings in the sports vision literature, offering important new insights into the domain specificity and complexity of visual function in sport.

Consistent with previous studies exploring physiological and anatomical correlates of physical activity, Ugurlu and Icel [20] reported that adult athletes display increased retinal microvascular density and thicker neural retinal layers compared to non-athletes, indicative of potential ocular health benefits of regular exercise. However, their cohort consisted of young, emmetropic adults with uniformly optimal VA, potentially obscuring subtle or discipline-specific visual differences. In contrast, our study encompassed a broader age range and included athletes with typical refractive variation and optical correction, revealing that the presumed superiority of visual acuity in athletes may not generalize across all sports. Notably, controls, despite being older on average, outperformed athletes on VA measures (Figure 1), highlighting that general physical activity does not necessarily translate to measurable advantages in standard clinical vision metrics. This emphasizes the need to interpret visual outcomes within specific sporting contexts and population characteristics.

A further dimension is illuminated by research into eSports participants [21], where mean refractive error and stereoacuity were within normative ranges, but higher accommodative lag and reduced tear breakup time were observed, attributed to intensive near-work demands. These findings align with our observation of largely normative clinical vision metrics across athlete groups, suggesting that sport participation alone does not inherently confer superior visual function. Instead, visual adaptations—beneficial or detrimental—appear to be shaped by each discipline’s perceptual and environmental demands. In line with this, our data indicate that certain sports may even harbor risks for specific visual challenges, such as accommodative fatigue or, as seen in roller derby athletes, potentially suboptimal VA due to ocular injuries, subclinical refractive changes, or inadequate correction.

More broadly, the lack of significant differences in peripheral vision and stereoacuity between groups aligns with contemporary research in interceptive and strategic sports. Studies by Gray [22], Kuo et al. [23], and Toole & Fogt [24] collectively suggest that elite athletic performance depends less on static clinical measures and more on the dynamic integration of visual–motor skills, anticipatory strategies, and environmental awareness. Our findings support this paradigm, showing that traditional tests may fail to capture the dynamic and context-specific visual skills critical for athletic performance. For instance, studies in baseball and badminton (Katsumata et al. [25]; Kato & Fukuda [26]; Ranganathan & Carlton [27]; Abernethy & Zawi [28]) have demonstrated that expert athletes rely on efficient gaze strategies and predictive cues rather than superior static visual metrics. Thus, the absence of group differences in clinical measures likely reflects the limits of static testing rather than the absence of functional visual expertise.

Despite these convergences, several unique distinctions merit consideration. Most notably, the inferior VA observed among roller derby athletes relative to both controls and other sports groups runs counter to expectations and warrants deeper exploration. Roller derby, as a full-contact, fast-paced sport, may select for or tolerate athletes with suboptimal visual acuity, perhaps compensated by superior spatial awareness, tactical anticipation, or resilience [29,30]. Alternatively, the demands and risks inherent to the sport, such as frequent physical contact, ocular trauma, or environmental exposure, could contribute to subtle reductions in VA not present in lower-contact or more visually demanding sports like basketball [31]. This discipline-specific pattern underscores the novelty of our findings and highlights the need for sport-tailored vision assessment and intervention.

From a methodological perspective, this study offers several notable strengths, including the inclusion of multiple sports alongside a well-matched non-athlete control group, which enabled more nuanced group comparisons beyond the typical single-sport designs. The application of rigorous statistical adjustment for confounders such as age adds robustness to the group comparisons, particularly for visual acuity. Comprehensive assessment protocols encompassing both clinical and functional measures allowed for a multifaceted view of visual performance, and the large effect sizes observed for visual acuity differences (e.g., Cohen’s d > 1) further emphasize the practical significance of these findings. Nevertheless, several limitations must be acknowledged. No formal a priori sample size calculation was conducted, as this was an exploratory, convenience-based study. The modest, convenience-based sample size may reduce statistical power and limit the generalizability of results, particularly for subgroup or sex-based analyses. The cross-sectional design precludes any conclusions regarding causality or longitudinal changes in visual function associated with training or sport participation. Reliance on standard clinical assessments, rather than dynamic or sport-specific visual–motor testing, may reduce sensitivity to detect functional adaptations relevant to real-game scenarios. Additionally, factors such as previous ocular injuries, compliance with optical correction, cumulative training load, or history of concussion were not systematically documented and may have influenced the observed results. Furthermore, as only objective non-cycloplegic autorefraction was performed, the absence of full subjective refraction may have introduced minor bias in the estimation of refractive status and its potential influence on visual acuity. Additionally, ocular alignment was assessed using the Hirschberg corneal reflex test rather than the cover test, the clinical gold standard. This choice allowed for objective and reproducible quantification of deviation across participants, but it may have reduced sensitivity for detecting subtle phorias. Given these sample size constraints, the findings should be interpreted as exploratory and hypothesis-generating rather than confirmatory. Future studies with larger and more diverse cohorts are warranted to validate and expand upon these preliminary results. Moreover, the present study focused exclusively on visual parameters; dynamic visual acuity and auditory function were not assessed. Including these measures in future work could provide a more comprehensive understanding of multisensory performance in athletes.

Building on these results, future research should prioritize longitudinal, multi-modal studies incorporating both static and dynamic visual assessments, ideally including wearable eye-tracking or motion capture in real-world sporting environments. Examining the intersection of visual performance, injury risk, and adaptation over time across different sports and age groups would clarify the mechanisms underlying sport-specific visual profiles. The inclusion of cognitive and perceptual–motor tasks, such as anticipation, decision-making under pressure, and visual search strategies, would provide a more holistic view of visual expertise in athletic contexts. In clinical and training settings, these findings highlight the importance of individualized vision screening and targeted intervention, particularly in sports where suboptimal acuity or unique visual demands may be prevalent.

## 5. Conclusions

This study challenges the commonly held belief that athletes inherently possess superior visual function compared to non-athletes. Our results indicate that there are some differences in visual acuity, with non-athlete controls and basketball players performing better and roller derby athletes performing worse. However, there were no significant group differences in peripheral vision, stereoacuity, ocular alignment, or refractive error. These findings suggest that participation in competitive team sports does not guarantee measurable advantages across conventional clinical vision metrics.

Importantly, these results align with current perspectives in the sports vision literature, which emphasize that high-level athletic performance relies more on dynamic perceptual–motor integration, anticipation, and context-specific visual strategies than on static clinical measures. The lack of differences in peripheral vision and stereoacuity between athletes and controls supports the notion that true expertise in sports vision is context-dependent and may not be detected by standard clinical tests alone. The lower visual acuity observed in roller derby athletes may reflect unique sport-specific risks, such as frequent physical contact or increased exposure to ocular trauma, as well as possible compensatory reliance on spatial awareness and tactical anticipation.

Overall, this work highlights the importance of individualized, sport-specific approaches to vision screening and training. Future research should incorporate ecologically valid, dynamic visual tasks and explore longitudinal changes in visual function among athletes across different sports. Moving beyond traditional clinical assessments will be crucial to fully understand and support visual performance in both athletes and non-athletes.

## Figures and Tables

**Table 1:** Descriptive statistics and group comparisons (ANOVA *p*-values) by sport group (mean ± SD, 95% CI).

Group	VA (logMAR)	Hirschberg (Δ)	Peripheral Vision (°)	Stereoacuity (arc sec)	Refractive Error (D)	Age (years)
Basketball	0.02 ± 0.05 [–0.02, 0.05]	5.79 ± 1.77 [4.53, 7.05]	37.0 ± 25.41 [18.8, 55.2]	96 ± 8.43 [89.97, 102.03]	–0.02 ± 0.52 [–0.40, 0.35]	20.0 ± 3.97 [17.16, 22.84]
Futsal	0.09 ± 0.10 [0.01, 0.16]	3.94 ± 1.93 [2.46, 5.42]	34.4 ± 14.24 [23.5, 45.4]	75.6 ± 19.44 [60.6, 90.5]	–0.83 ± 0.56 [–1.26, –0.40]	24.9 ± 4.40 [21.51, 28.27]
Hockey	0.07 ± 0.13 [–0.02, 0.15]	4.40 ± 1.90 [3.12, 5.68]	26.4 ± 18.04 [14.24, 38.48]	79.1 ± 54.86 [42.24, 115.94]	–0.26 ± 1.32 [–1.15, 0.63]	27.2 ± 5.44 [23.53, 30.83]
Roller Derby	0.16 ± 0.12 [0.08, 0.24]	3.65 ± 1.89 [2.38, 4.92]	24.2 ± 29.94 [4.07, 44.30]	66.4 ± 50.25 [32.60, 100.12]	–0.57 ± 0.34 [–0.79, –0.34]	33.6 ± 3.32 [31.40, 35.87]
Control	–0.00 ± 0.08 [–0.06, 0.05]	5.17 ± 1.76 [3.99, 6.36]	17.2 ± 21.06 [3.04, 31.33]	123.6 ± 109.11 [50.33, 196.94]	–0.66 ± 1.71 [–1.81, 0.49]	35.6 ± 10.88 [28.32, 42.95]
*p*-value	**0.003**	0.24	0.41	0.36	0.58	**<0.001**

Note: *p*-values obtained from one-way ANOVA across sport groups. Statistically significant results (*p* < 0.05) are shown in bold. Post hoc Tukey’s pairwise comparisons indicated that visual acuity was significantly different between roller derby and both control and basketball groups.

## Data Availability

The original contributions presented in this study are included in the article. Further inquiries can be directed to the corresponding author.
